# Rapid biotransformation of STW 5 constituents by human gut microbiome from IBS- and non-IBS donors

**DOI:** 10.1128/spectrum.04031-23

**Published:** 2024-05-13

**Authors:** Timo A. Thumann, Eva-Maria Pferschy-Wenzig, Christina Kumpitsch, Stefanie Duller, Christoph Högenauer, Patrizia Kump, Heba Aziz-Kalbhenn, Ramy M. Ammar, Sabine Rabini, Christine Moissl-Eichinger, Rudolf Bauer

**Affiliations:** 1 Department of Pharmacognosy, Institute of Pharmaceutical Sciences, University of Graz, Graz, Austria; 2 BioTechMed, Graz, Austria; 3 Diagnostic and Research Institute of Hygiene, Microbiology and Environmental Medicine, Medical University of Graz, Graz, Austria; 4 Department of Internal Medicine, Medical University of Graz, Graz, Austria; 5 Steigerwald Arzneimittelwerk GmbH, Bayer Consumer Health, Darmstadt, Germany; 6 Department of Pharmacology, Faculty of Pharmacy, Kafrelsheikh University, Kafrelsheikh, Egypt; Lerner Research Institute, Cleveland, Ohio, USA

**Keywords:** STW 5, IBS, next-generation sequencing, microbiome, metabolomics, UHPLC-HRMS

## Abstract

STW 5, a blend of nine medicinal plant extracts, exhibits promising efficacy in treating functional gastrointestinal disorders, notably irritable bowel syndrome (IBS). Nonetheless, its effects on the gastrointestinal microbiome and the role of microbiota on the conversion of its constituents are still largely unexplored. This study employed an experimental *ex vivo* model to investigate STW 5’s differential effects on fecal microbial communities and metabolite production in samples from individuals with and without IBS. Using 560 fecal microcosms (IBS patients, *n* = 6; healthy controls, *n* = 10), we evaluated the influence of pre-digested STW 5 and controls on microbial and metabolite composition at time points 0, 0.5, 4, and 24 h. Our findings demonstrate the potential of this *ex vivo* platform to analyze herbal medicine turnover within 4 h with minimal microbiome shifts due to abiotic factors. While only minor taxonomic disparities were noted between IBS- and non-IBS samples and upon treatment with STW 5, rapid metabolic turnover of STW 5 components into specific degradation products, such as 18β-glycyrrhetinic acid, davidigenin, herniarin, 3-(3-hydroxyphenyl)propanoic acid, and 3-(2-hydroxy-4-methoxyphenyl)propanoic acid occurred. For davidigenin, 3-(3-hydroxyphenyl)propanoic acid and 18β-glycyrrhetinic acid, anti-inflammatory, cytoprotective, or spasmolytic activities have been previously described. Notably, the microbiome-driven metabolic transformation did not induce a global microbiome shift, and the detected metabolites were minimally linked to specific taxa. Observed biotransformations were independent of IBS diagnosis, suggesting potential benefits for IBS patients from biotransformation products of STW 5.

**IMPORTANCE:**

STW 5 is an herbal medicinal product with proven clinical efficacy in the treatment of functional gastrointestinal disorders, like functional dyspepsia and irritable bowel syndrome (IBS). The effects of STW 5 on fecal microbial communities and metabolite production effects have been studied in an experimental model with fecal samples from individuals with and without IBS. While only minor taxonomic disparities were noted between IBS- and non-IBS samples and upon treatment with STW 5, rapid metabolic turnover of STW 5 components into specific degradation products with reported anti-inflammatory, cytoprotective, or spasmolytic activities was observed, which may be relevant for the pharmacological activity of STW 5.

## INTRODUCTION

Irritable bowel syndrome (IBS) is a functional bowel disorder. The reported prevalence estimates for which are very broad, ranging from 1.1% to 45% (1). The clinical picture is characterized by recurrent abdominal pain, disturbed bowel habits (constipation and/or diarrhea), and symptoms such as flatulence and bloating (2–4). Although the etiology of IBS remains poorly understood, several pathophysiological mechanisms appear to be involved in triggering IBS symptoms (3–5), including genetic factors, dysregulation of the immune system, gut-brain miscommunication, chronic infections, food hypersensitivity, and dysbiosis of the gut microbiome (3, 4, 6).

The gastrointestinal tract (GIT) is the spot of highest microbial abundance and diversity in the human body. The GIT microbiome fulfills important tasks for the physiology and health homeostasis of its human host (7) and includes mostly bacterial taxa like *Bacteroides*, *Faecalibacterium*, Ruminococcaceae sp., but also archaea (e.g., *Methanobrevibacter* species), viruses and fungi (8). However, microbial community composition is subject to many factors, such as dietary habits, health status, and medication (9, 10). This variation also applies to IBS in which some studies have found the GIT microbiome to differ from that of healthy individuals in terms of microbial composition (shown by 16S rRNA gene sequencing), metabolites (assessed by metabolomics), and metabolic pathways (based on shotgun metagenomics) (11). The degree of alteration is mainly associated with the severity of the disease as highlighted earlier (12). These differences between the gut microbiome profiles of IBS patients and healthy controls were also discussed in a recent systematic review (13). Enterobacteriaceae, Lactobacillaceae, and *Bacteroides* taxa were increased in IBS patients, whereas *Faecalibacterium* and *Bifidobacterium* were reduced compared to controls (13).

Several studies have already shown a positive effect of herbal medicinal products on dysbiotic GIT microbiota observed in different disease states like colorectal cancer (14) and obesity (15). On the one hand, herbal products such as licorice—a component of STW 5—can modulate the composition of the GIT microbiome (16), exerting prebiotic-like effects (17). On the other hand, the GIT microbiota can biotransform herbal medicines to produce new absorbable small molecules that can have pharmacological effects either in the gut or systemically (18, 19). Examples of such molecules are urolithins that are formed upon gut microbial metabolism of ellagitannins (20), phenylvalerolactones or phenolic acids deriving from biotransformation of procyanidins or flavonoids (18, 21), or deglycosylated metabolites from triterpene glycoside metabolism, such as ginsenoside-derived compound K (22) or glycyrrhizic acid-derived glycyrrhetinic acid (23). The interactions between medicinal plants commonly used for gastrointestinal disorders and the human gastrointestinal microbiome were recently reviewed. Several of these plants have been found to influence microbial community composition, exerting prebiotic-like or antimicrobial effects, or to modulate microbial production of short-chain fatty acids. Also, many of their constituents have been shown to be subject of microbial biotransformation, leading to the production of potentially bioactive metabolites (24).

STW 5 is a herbal medicinal product with proven clinical efficacy in the treatment of functional gastrointestinal disorders, like functional dyspepsia and IBS (25, 26). The multi-target pharmacological effect of STW 5 has been confirmed in several *in vitro* and *in vivo* studies (27). These potential benefits of STW 5 include an anti-inflammatory effect, modulatory properties on ion channels, and a region-specific eukinetic effect on gut motoric activity. Overall, this contributes to an improvement in gastrointestinal symptoms by restoring impaired intestinal permeability and reducing visceral hypersensitivity (28, 29). In a recent study, inflammatory processes and gut microbiome dysbiosis could be largely prevented by STW 5 administration in induced ulcerative colitis mouse models (27).

STW 5 contains a combination of nine herbal extracts. The major constituents of this herbal mixture are triterpene saponins, flavonoid glycosides, cinnamic acid derivatives, and alkaloids (30). Our previous study showed that the majority of STW 5 constituents are not or only partially degraded in an *in vitro* static digestion model simulating conditions in the oral cavity, stomach, and small intestine. This indicated that the constituents could reach the human colon and interact with the gut microbiota if not absorbed in the small intestine (30); moreover, a recent study has shown that STW 5-II, a sister formulation containing six of the nine herbal extracts present in STW 5, interacts with human fecal microbiota in an *in vitro* short-term colonic model (31).

In this study, an experimental *ex vivo* platform was used to (i) determine the modulation and putative differential effects of STW 5 on the fecal microbial community of individuals with and without IBS and (ii) analyze shifts in the complex metabolic profile of STW 5 constituents caused by microbial biotransformation. This possible two-way interaction was investigated by incubating STW 5 with fecal microbial samples of individuals with and without IBS.

## RESULTS

### Study design

To gain preliminary insights into the bilateral interaction between the herbal preparation STW 5 and the human gut microbiome, human fecal suspensions (HFSs) from 10 subjects without IBS (healthy, non-IBS) and 6 subjects with IBS (predominant diarrhea type according to ROME IV criteria) were incubated *ex vivo* with *in vitro* pre-digested STW 5 or a control (VEH) sample for up to 24 h. VEH contained all reagents of *in vitro* pre-digestion, but no STW 5. Pre-digestion was performed to mimic the passage of STW 5 constituents through the upper digestive tract (see Materials and Methods for more details).

**Fig 1:**
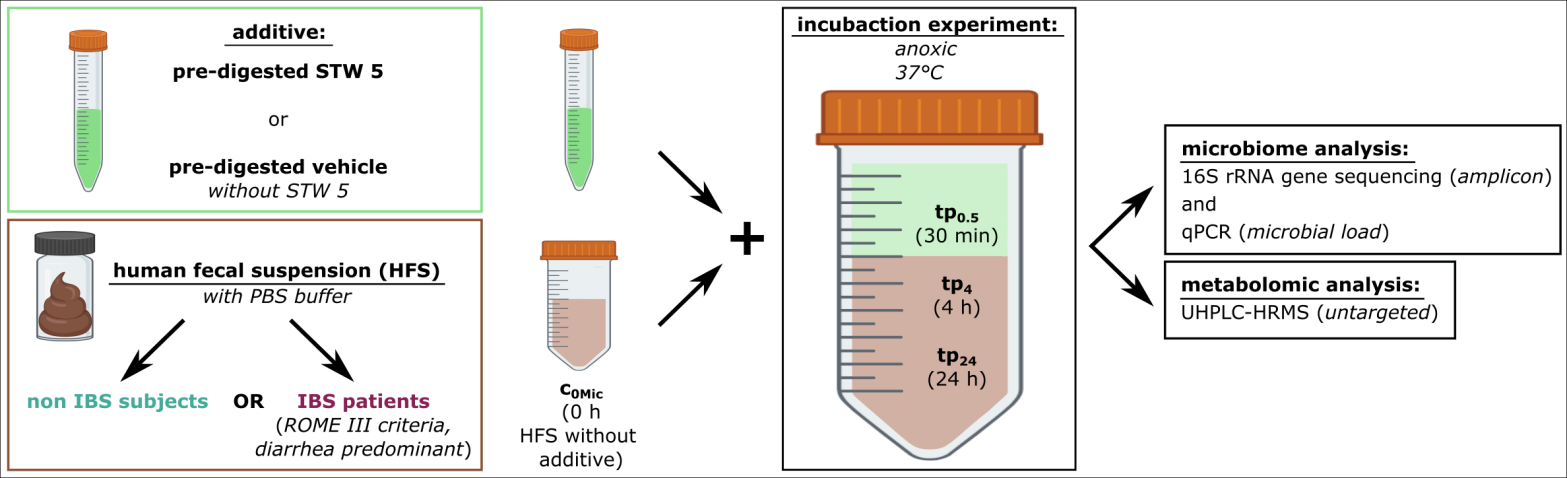
Study design. Human fecal suspensions (HFSs) of healthy subjects (non-IBS) or IBS patients were mixed with pre-digested STW 5 or pre-digested vehicle (VEH), respectively. Per participant and condition, five replicates were set up. Samples were collected before incubation with the specific additive and 30 min, 4 h, and 24 h after addition at 37°C under anoxic conditions. Microbiome and metabolomic analysis were performed with all samples and replicates.

In total, 560 samples were collected at time point 0 (c_0Mic_, before addition of pre-digested STW 5 or VEH; *n* = 80), after 30 min (tp_0.5_; *n* = 160), 4 h (tp_4_; *n* = 160), and 24 h (tp_24_; *n* = 160) of incubation (for detailed information on the sample size see Fig. S1). Potential changes in microbial composition due to STW 5 at all time points were assessed by amplicon sequencing of the 16S rRNA gene (V4 region). In addition, the microbial load was quantified by qPCR and in order to assess microbial metabolism of STW 5 constituents, metabolite profile changes in the incubates were detected by untargeted ultra high-performance liquid chromatography-mass spectrometry (UHPLC-HRMS) analysis (Fig. 1).

### Quality control of the amplicon data of the HFS samples

An analytical sample size flowchart of the study is provided in Fig. S1. Overall, 560 samples from 16 donors were processed within this study. For quality control, reads obtained from the negative/process controls were subtracted, and samples that did not meet the specified quality criteria (e.g., with respect to number of reads) were excluded. SRS (Scaling with Ranked Subsampling) was used to normalize the data for all further analysis. In this step, additional three samples did not meet the minimal sampling depth of 10,000 and were excluded. The final data set included 552 samples with 4,022 RSVs and 5,520,000 reads for further analyses (for detailed information on the filtering, see Materials and Methods).

### Baseline microbial signatures do not differ between fecal samples from non-IBS and IBS donors

To initially assess the original microbial community composition and potential differences between samples from healthy and IBS donors, c_0Mic_ samples (HFSs without any treatment) were analyzed.

**Fig 2:**
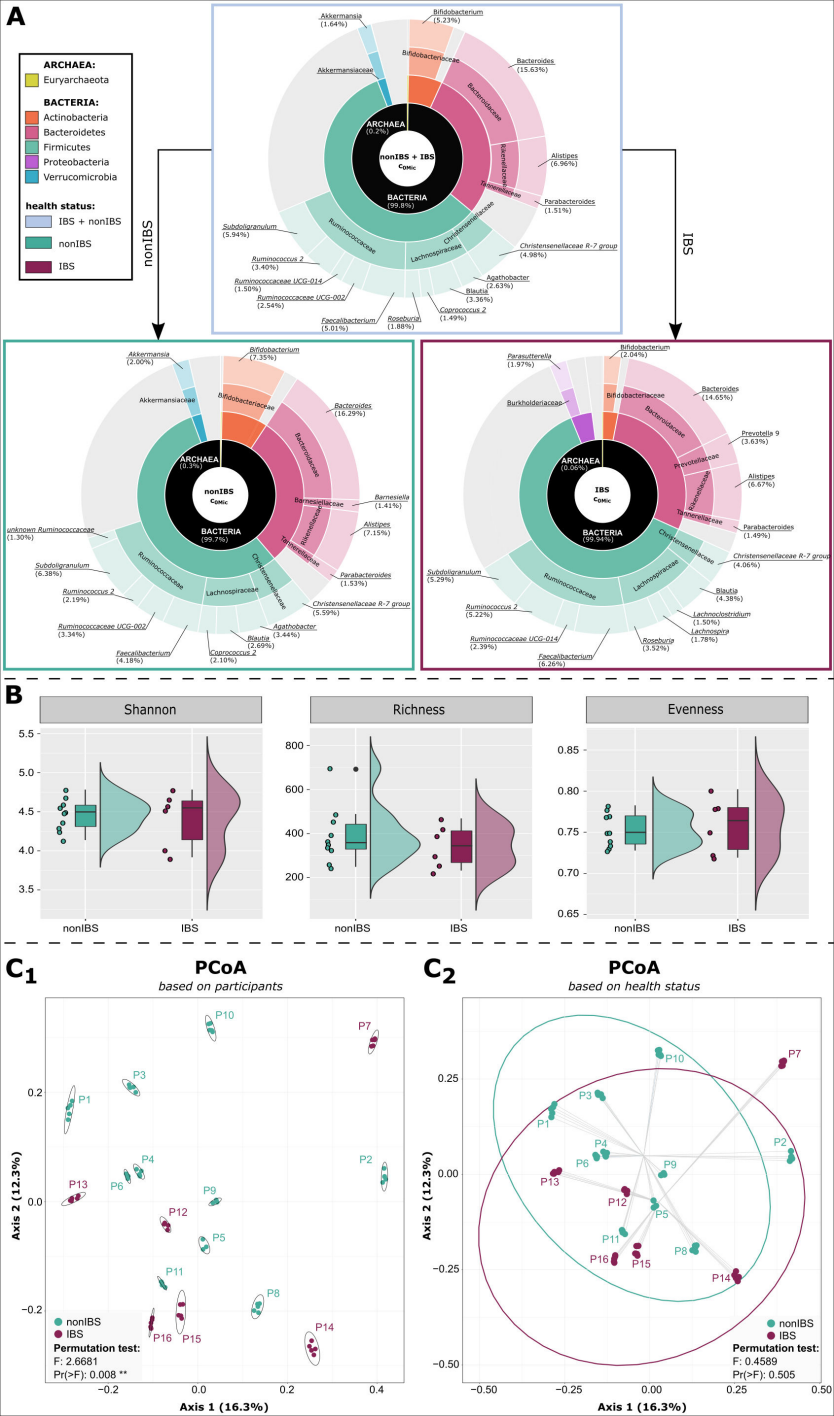
Microbial composition of non-IBS and IBS HFS at baseline (c_0Mic_). (A) Pie charts of the 15 most abundant genera found in non-IBS and IBS samples, respectively. (B) Alpha diversity of the gut microbiome is not affected by health status. The means of the participant replicates were used for the analysis to deal with the technical replicates (Health status: unpaired; Shannon and Evenness: parametric → *t* test; Richness: non-parametric → Mann-Whitney *U* test; Shannon: 0.739; Richness: 0.515; Evenness: 0.484). (C) Microbial composition clustered based on participants rather than health status in a PCoA plot. (C_1_) based on participants (C_2_) based on health status. All plots were generated using the SRS-normalized data (baseline c_0Mic_).

Fecal samples from both groups at time point c_0Mic_ were predominated by similar taxa. Firmicutes (56% non-IBS; 62% IBS) and Bacteroidetes (30% non-IBS; 29% IBS) dominated both groups at phylum level with either Actinobacteria (7% non-IBS; 3% IBS) or Proteobacteria (3% non-IBS; 4% IBS) on third position in non-IBS and IBS, respectively. Genera such as *Bacteroides* (16% non-IBS; 15% IBS), *Subdoligranulum* (6% non-IBS; 5% IBS), *Bifidobacterium* (7% non-IBS; 2% IBS), *Alistipes* (7% non-IBS; 7% IBS), or *Faecalibacterium* (4% non-IBS; 6% IBS) were found among the most abundant taxa in the whole data set (Fig. 2A).

In general, the summarized replicates per healthy subjects did not differ significantly in alpha diversity compared to IBS patients; however, the IBS group seemed to be more even (Fig. 2B; Table S1A). The five technical replicates of each participant formed a distinct cluster, indicating low intra-sample variability and high stability of the applied experimental settings. However, no separate clustering was observed based on health status (IBS, non-IBS) in the PCoA plot (Fig. 2C).

Discriminatory analysis (MaAsLin2_SR) between IBS and non-IBS in c_0Mic_ samples revealed some taxa with higher prevalence (*P* value < 0.05) in one or the other health condition. Taxa belonging to the phylum Firmicutes, in particular to the family Lachnospiraceae, were predominantly found in HFS from IBS patients (Table S1D). Two genera, namely *Lachnoclostridium* (*P* = 0.02) and *Roseburia* (*P* = 0.005), were among the 15 most abundant taxa found in IBS samples (Fig. 2A). Putative biomarkers for non-IBS HFS included some taxa of the families Ruminococcaceae or Rikenellaceae. Nevertheless, none of the mentioned taxa remained significant after correction (*q* value) (Table S1D).

### The experimental setup allows an unbiased 4-h window for *ex vivo* analysis of effects of STW 5 on fecal samples

**Fig 3:**
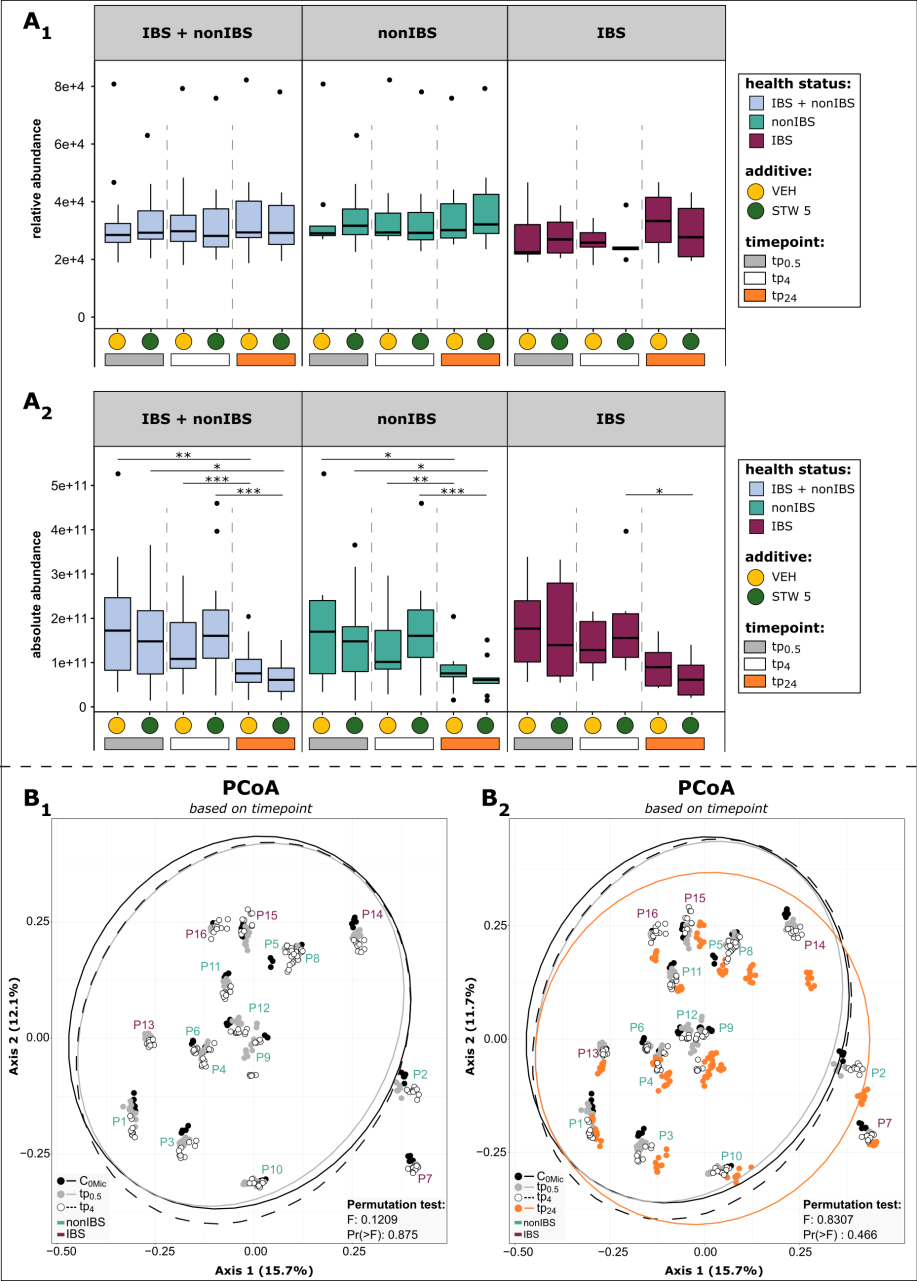
The duration of incubation did not drastically alter the microbial load and composition up to tp_4_. (A_1_) the relative abundance of 16S rRNA gene sequences and (A_2_) absolute abundance (per 1 g stool) over time. The means of alpha diversity measures were used for the analysis to deal with the technical replicates (statistics based on time points (paired) → Friedman test or paired *t* test; based on additive (unpaired) → Mann-Whitney *U* test or unpaired *t* test; non-SRS normalized data set; for *P* values, see Table S1). (B) Microbial community composition did change not based on participant groups (B_1_) but showed a slight shift based on time points after 24 h of incubation (B_2_) (SRS normalized data set).

Although the relative abundance of the microbial taxa was quite stable during incubation, the absolute abundance determined by qPCR showed significant differences over time (Fig. 3A1 and A2). The copy number of the 16S rRNA gene per gram of stool increased slightly but not significantly after 4 h of incubation, while the microbial load tended to decrease in the vehicle-treated samples. However, after 24 h of incubation, a significant decrease in the microbial load was observed both in STW 5- and vehicle-treated samples (Fig. 3A2, for *P* values, see Table S1B and C). Furthermore, the microbial community of t_24_ clustered separately from c_0Mic_, tp_0.5_, and tp_4_ of the replicates of the same participant in the PCoA plot (Fig. 3B1 and B2). These differences in microbial load and community composition could be explained by the depletion of nutrients or other dynamics occurring in the static batch cultures after an extended period of time.

It shall be noted that the described trends were observable in all groups (non-IBS, IBS, and combined). Therefore, in the following, to be maximally conservative, we focused on further analysis of the microbiome at tp_0.5_ and tp_4_ to avoid bias in interpretation.

### STW 5 does not affect overall alpha and beta diversity, but reveals potential effects on specific microbial taxa

To evaluate the changes in microbial composition induced by *in vitro* pre-digested STW 5 compared to *in vitro* pre-digested vehicle (VEH; details are given in Materials and Methods) in samples from IBS patients and healthy controls, the relative microbial abundance in tp_0.5_ and tp_4_ samples was subjected to further statistical analysis.

**Fig 4:**
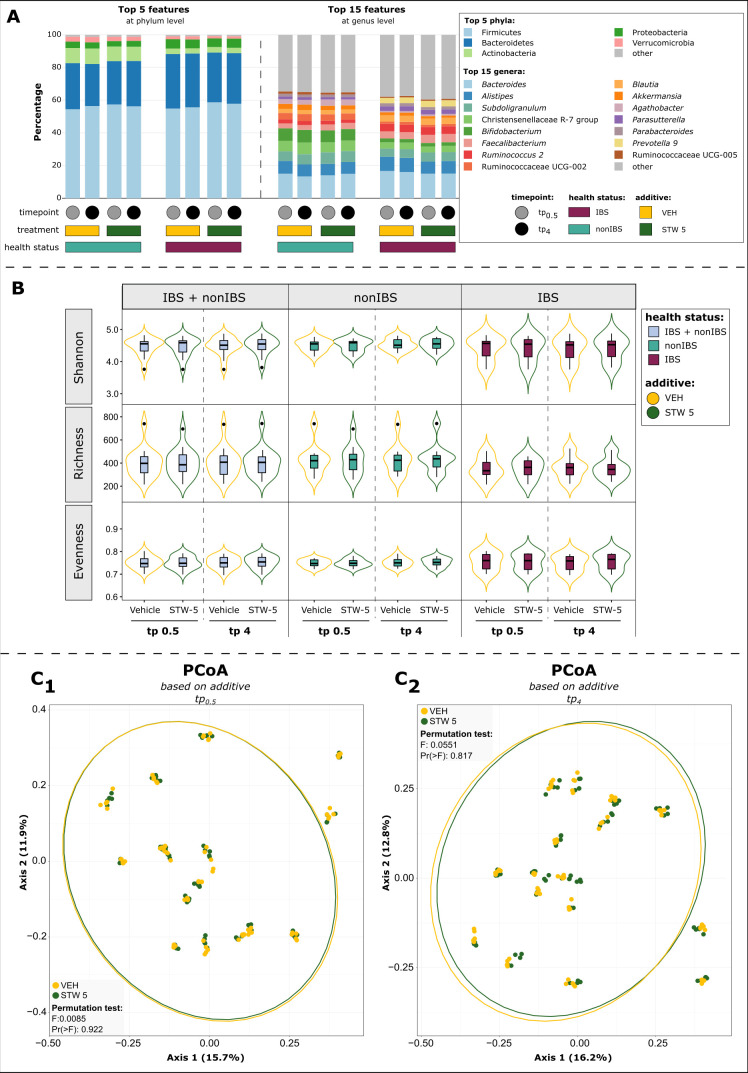
STW 5 only influenced microbial community on the taxa-level in both health groups compared to VEH. (A) Bar plot showing the most abundant taxa at phylum and genus level separated by health status, treatment, and time. (B) Alpha diversity indices did not reveal significant differences. The means of the participant replicates were used for the analysis to deal with the technical replicates (unpaired: STW 5 vs VEH; paired: time points; for *P* values, see Table S1B and C). (C) The additives did not shift the microbial community as a whole in one or the other direction—neither after (C_1_) 30 min nor (C_2_) 4 h of incubation.

In comparison to the previous analyses at baseline c_0Mic_ (Fig. 2A), the predominant phyla and genera remained the same over time (tp_0.5_ and tp_4_) independently of the used treatment (STW 5 and VEH), indicating that STW 5 did not massively influence microbiome composition (Fig. 4A). Moreover, the different treatments had no influence on any of the microbial alpha diversity indices at tp_0.5_ and tp_4_ in both groups (Fig. 4B; for *P*-values see Table S1A). In addition, the overall structure of the microbial community was not significantly altered by STW 5 compared to VEH addition at any time point (Fig. 4C).

In general, taxonomic differences (*P* < 0.01) were detectable between non-IBS and IBS samples based on the two treatment options. A full overview is provided in Fig. S2A. *Ruminococcus 1* as well as Actinobacteria and *Anaerotruncus* distinguished the VEH-treated HFS-samples of non-IBS and IBS donors at tp_0.5_ and tp_4_, respectively. STW 5 addition led to higher amounts of *Roseburia* in IBS and *Anaerotruncus* signatures in non-IBS samples at tp_4_.

*Ruminococcus* 1 and *Roseburia* were found to be decreased (*P* < 0.01) in non-IBS and IBS samples, respectively, under the influence of STW 5 (Fig. S2A, for *P* values, see Table S1D). It shall be noted, that most of these variations did not withstand *P* value corrections, so they have to be considered with caution.

Since the health status did not significantly (*q* < 0.05) affect microbial community members at c_0Mic_ or other time points (tp_0.5_, tp_4_) (Fig. S2A, for *P* values, see Table S1D), further analyses of microbial biomarkers for STW 5 and VEH, were performed using the combined data set (non-IBS + IBS samples).

Interestingly, the significant associations (*P* < 0.01) observed in HFS at phylum level (a higher relative abundance of Proteobacteria and Bacteroidetes in vehicle-treated samples and of Firmicutes in STW 5-treated samples) at tp_0.5_ changed to the opposite after 4 h of incubation, indicating a potential benefit for Proteobacteria and Bacteroidetes by STW 5 over the course of time (Fig. S2B, for *P* values, see Table S1D). At genus level, just one taxon, namely unknown Rhodospirillales (tp_4_) was found to be associated with STW 5, whereas VEH-biomarkers included more taxa at both time points such as *Sutterella* or *Victivallis* at tp_0.5_ and *Ruminococcaceae UCG-013* or *Ruminiclostridium 5* at tp_4_ (Fig. S2B, for *P* values, see Table S1D). This could indicate a potential inhibition of those taxa by STW 5.

Analysis over time revealed many time-dependent taxonomic associations in STW 5 and VEH-treated HFS. However, similar trends were observed for both additive groups at the selected time points (e.g., variations in *Agathobacter*, *Faecalibacterium* and *Roseburia*, *Akkermansia*, *Alistipes*, and *Lachnoclostridium*). Nevertheless, specific taxa (such as *Methanobrevibacter*, *Bifidobacterium* for VEH or *Sellimonas* and *Anaerostipes* for STW 5) were associated with just one additive group (Fig. S2B, for *P* values, see Table S1D).

Since our results indicated enhanced levels of phylum Bacteroidetes und RSV *Bacteroides* in STW 5-treated samples compared to VEH-treated samples at tp_4_, we checked potential biomarkers belonging to the phylum Bacteroidetes. Analysis based on RSV level revealed three Bacteroidetes RSVs (*P* < 0.01) to be associated with VEH at tp_0.5_ and three with STW 5 at tp_4_. Additionally, more RSVs were defined as biomarkers in STW 5 at tp_4_ compared to tp_0.5_ and vice versa in VEH. In general, the significantly different RSVs found in the comparisons belonged to similar taxa including *Bacteroides*, *Parasutterella*, or *Alistipes* (Fig. S2C, for *P* values, see Table S1D).

For completeness, the separated data sets (non-IBS and IBS) showed similar results at phylum and genus levels compared to the combined one. (Fig. S3A and B, for *P* values, see Table S1D). Again, the significant associations (*q* < 0.05) observed in non-IBS HFS at phylum level at tp_0.5_ changed to the opposite after 4 h of incubation. However, in IBS HFS, the difference was not as prominent. Just one characteristic taxon for VEH, namely the phylum Proteobacteria (*q* = 0.004), was found at tp_0.5_. No further significant differences could be observed at tp_0.5_ nor tp_4_ (Fig. S3A and B for *P* values, see Table S1D).

Mainly, trends (*P* < 0.01) were detectable at genus level based on the treatments in both IBS- and non-IBS-donor samples. Several taxa were characteristic of VEH-treated samples, but fewer microbial signatures were associated with STW 5. Except for unknown Rhodospirillales (VEH biomarker at tp_0.5_), no genus was significantly (*q* < 0.05) associated with one or the other additive (Fig. S3A and B, for *P* values, see Table S1D).

### Human gut microbiota rapidly biotransform STW 5 constituents into lower molecular-weight metabolites

The microbial turnover of the previously identified major STW 5 constituents (30) and the formation of metabolites were analyzed by UHPLC-HRMS.

**Fig 5:**
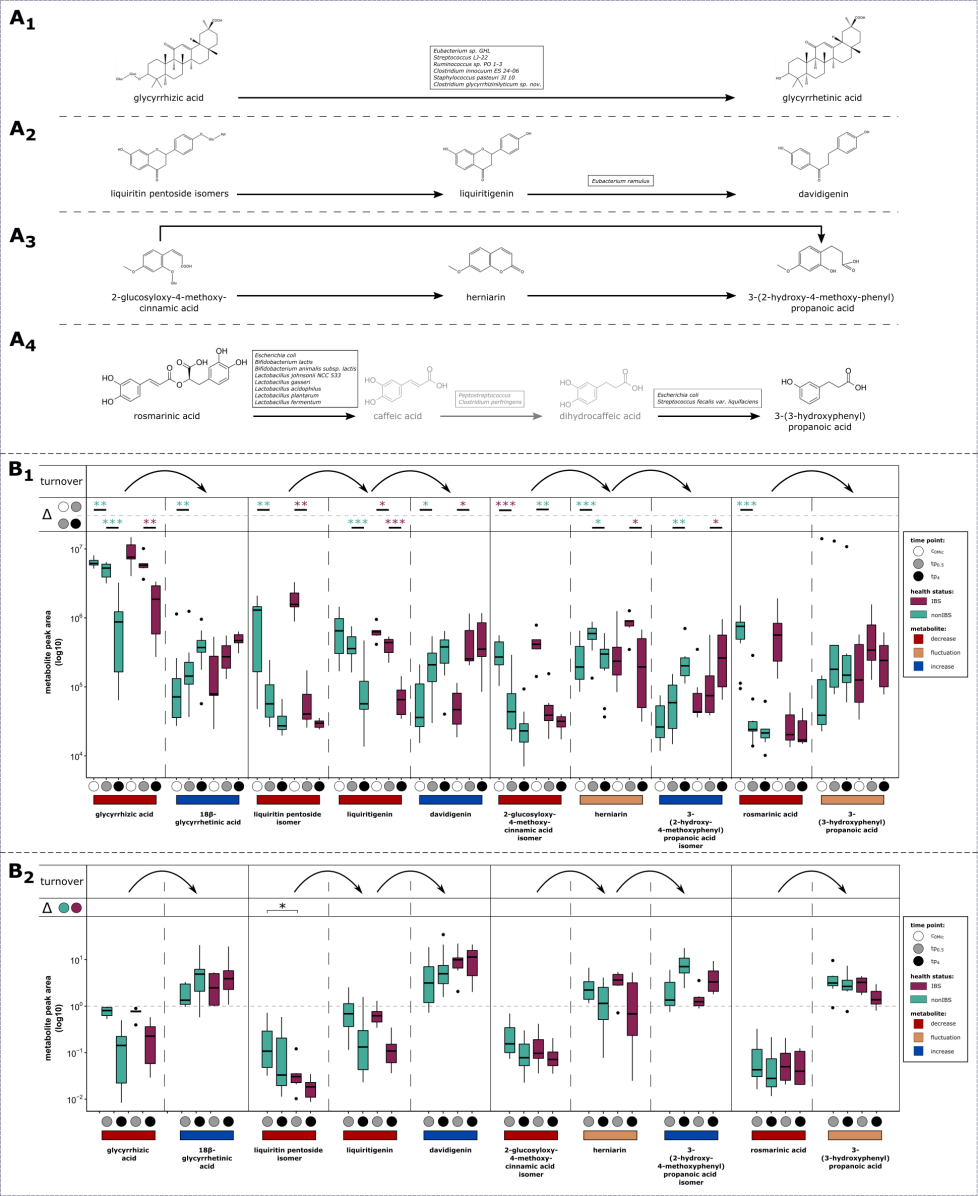
(A) Proposed biotransformation pathways of STW 5 constituents by gut microbiota. (A_1_) Glycyrrhizic acid can potentially be deglycosylated by several bacterial taxa (32–36). (A_2_) Liquiritin pentoside and liquiritigenin. Deglycosylation of flavonoids is accomplished by a wide range of microbial taxa (37). Conversion of liquiritigenin to davidigenin has been described for *Eubacterium ramulus* (38). (A_3_) 2-glucosyloxy-4-methoxycinnamic acid isomer and herniarin. (A_4_) Ester hydrolysis of rosmarinic acid (39–41) and subsequent transformation of caffeic acid into 3-(3-hydroxyphenyl) propanoic acid via dihydrocaffeic acid (42) can be induced by the listed bacterial taxa (caffeic acid and dihydrocaffeic acid intermediates have not been detected in this study) (B) Peak areas of STW 5 constituents and their turnover products over time in HFS. (B_1_) Peak areas compared over time in non-IBS and IBS, respectively. (B_2_) Ratios (tp_0.5_/c_0Met_ and tp_4_/c0_Met_) were used to compare metabolite peak areas based on health status. Horizontal lines in the boxes represent median peak areas. Average peak area per metabolite for each participant was calculated to minimize the bias by using replicates (statistical tests: paired samples (over time): Wilcoxon and paired *t* test; unpaired samples (health status): Mann-Whitney *U* test or unpaired *t* test; for *P* values, see Table S1E).

The major STW 5 constituents included triterpene saponins like glycyrrhizic acid, cinnamic acid derivatives like rosmarinic acid and 2-glucosyloxy-4-methoxycinnamic acid isomers, and flavonoids such as liquiritin pentosides and their aglycone liquiritigenin (Fig. 5A).

These compounds were consistently detectable at baseline, i.e., in the c_0Met_ samples (Annotation, see Table S2). Upon incubation with HFS, the levels of these compounds were found to consistently decrease over time, while they remained unchanged in the microbiome-free control samples. The levels of the phenolic compounds rosmarinic acid, 2-glucosyloxy-4-methoxycinnamic acid isomer, and liquiritin pentoside decreased very fast, with median ratios compared to c_0Met_ below 0.5 already after 0.5 h of incubation. Liquiritigenin was initially contained in STW 5, but obviously also intermittently formed by deglycosylation of liquiritin pentoside (see Fig. 5B; for *P* values, see Table S1E). Therefore, its median ratio compared to c_0Met_ decreased below 0.5 only after 4 h of incubation. The triterpene glycoside glycyrrhizic acid was degraded slower than the phenolic compounds, reaching a median ratio below 0.5 compared to that present in c_0Met_ only after 4 h of incubation.

This decrease in STW 5 constituents was accompanied by an increase in the levels of their metabolites. As major metabolites, 18β-glycyrrhetinic acid, davidigenin, herniarin, 3-(3-hydroxyphenyl)propanoic acid, and 3-(2-hydroxy-4-methoxyphenyl)propanoic acid were annotated (Fig. 5B; for *P* values, see Table S1E). While 18β-glycyrrhetinic acid, davidigenin, herniarin, and 3-(3-hydroxyphenyl)propanoic acid accumulated over incubation time, the levels of herniarin initially increased before decreasing again. This indicates that herniarin is further biotransformed to other products, most likely 3-(2-hydroxy-4-methoxyphenyl) propanoic acid. The pathways proposed for gut microbial formation of intermediates and final metabolites are shown in Fig. 5A.

As far as the impact of health status on the turnover of STW 5 constituents is concerned, in general, similar trends were observed for metabolism of STW 5 constituents in IBS and non-IBS samples (Fig. 5B1; for *P* values, see Table S1E). In case of HFS from non-IBS subjects, the constituents glycyrrhizic acid, 2-glucosyloxy-4-methoxy-cinnamic acid isomer, and rosmarinic acid significantly decreased from c_0Met_ to tp_0.5_, whereas glycyrrhizic acid was significantly decreased only after tp_4_ in IBS-HFS (Fig. 5B1; for *P* values, see Table S1E). Nevertheless, only the turnover ratio (tp_0.5_/c_0Met_) of liquiritin pentoside isomer at tp_0.5_ was found to be significantly different between the non-IBS and IBS samples (Fig. 5B2; for *P* values, see Table S1E). Apart from that, no statistically significant differences of any turnover product could be observed based on health status (Fig. 5B; for *P* values, see Table S1E).

### The composition of the microbiome reflects the turnover of STW 5 compounds only to a small extent

In this study, the turnover of five major STW 5 constituents over time was observed. This metabolism happened after administration to HFS samples, indicating an active role of the microbes present in stool in the turnover. To identify the microbes that are potential candidates for metabolism, correlation analysis was performed based on our collected metabolome and microbiome data.

**Fig 6:**
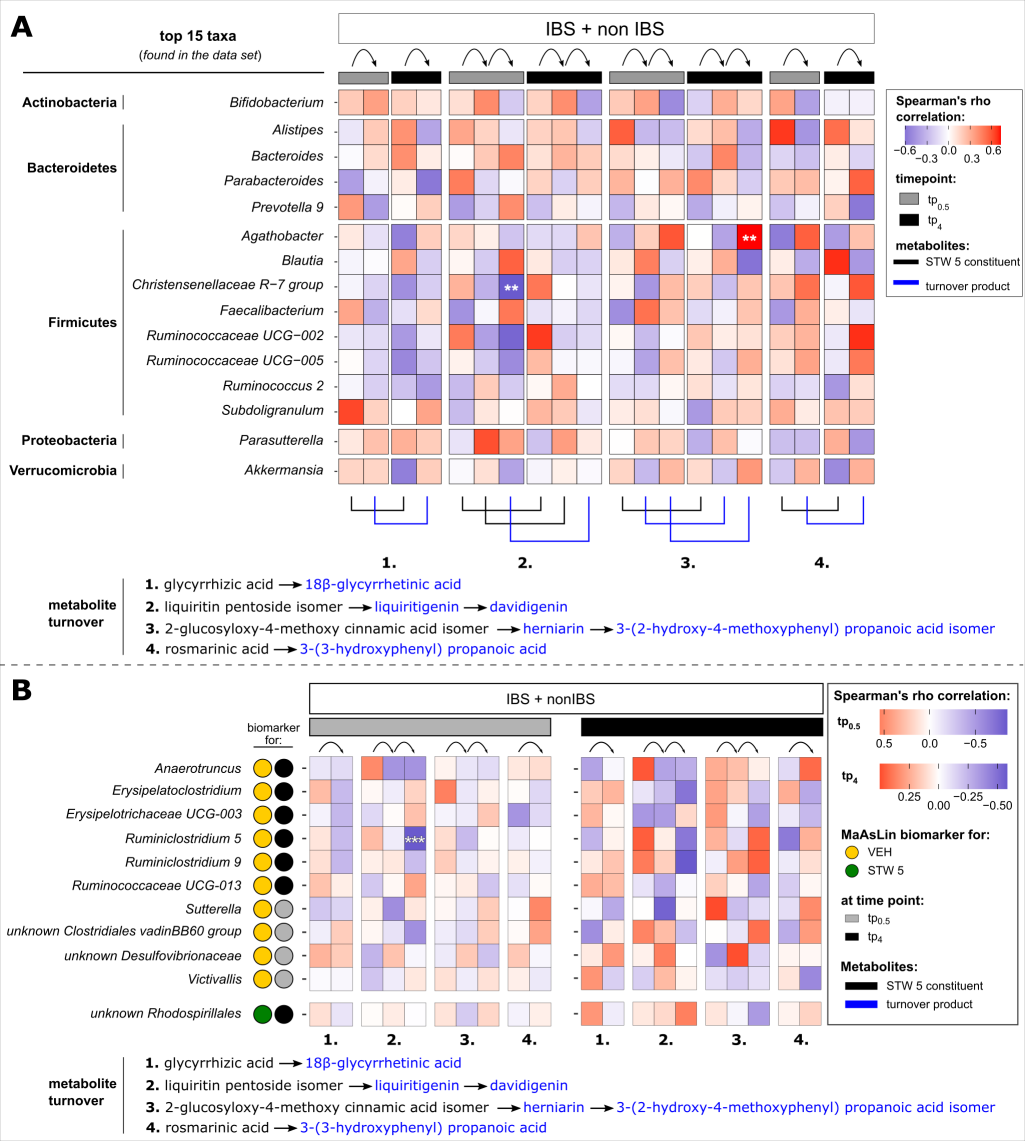
Spearman’s rho correlation between metabolites and top 15 markers (**A**) and biomarkers for VEH and STW 5 (**B**), respectively, found in the combined data set.

When considering the dominant taxa, just two correlations remained significant after multiple testing correction (*q* < 0.05). *Agathobacter* and *Christensenellaceae-R7 group* were correlated with one STW 5 turnover product—2-(2-hydroxy-4-methoxyphenyl) propionic acid isomer at tp_4_ and davidigenin at tp_0.5_, respectively (Fig. 6A). Both taxa could not be identified as taxonomic biomarkers for one or the other additive by discriminative analysis (see above). However, *Agathobacter* tended to be more abundant in tp_0.5_ HFS compared to tp_4_ (Fig. S2 and S3).

A possible contribution to the metabolic turnover of the STW 5 constituents was also investigated based on the identified biomarkers for STW 5 HFS (Fig. 6B). The only taxon that was linked to STW 5 administration did not correlate significantly with any of the observed metabolites in the HFS. In the case of VEH biomarkers, just *Ruminiclostridium 5* was found to be negatively associated with davidigenin (Fig. 6B).

## DISCUSSION

This study investigated the possible interactions between pre-digested STW 5 and human gut microbiota in an experimental *ex vivo* platform. The main findings can be summarized as follows:

1. The established *ex vivo* analysis platform showed a high applicability to analyze the turnover of herbal medicines within 4 h without major shifts in the microbiome caused by abiotic factors. The microbial load of HFS remained stable up to 4 h after incubation; however, a drastic decrease was detectable in both treatment groups after 24 h of incubation, most likely due to nutrient deprivation.
2. Minor differences were observed between the microbial composition of HFS treated with pre-digested VEH or STW 5.
3. When incubated with HFS, very fast metabolic turnover of STW 5 constituents into their respective metabolites was observed.
4. The metabolic turnover was not well mirrored by an extensive, statistically significant shift of the overall microbiome or microbial taxa.
5. Non-IBS and IBS microbiomes, in our study, were not significantly different from each other and did not reveal significant differences in the capability for metabolic transformation of STW 5 constituents (except for liquiritin-pentoside isomers).

In the applied experimental *ex vivo* platform, viable/intact microbial biomass (PMA-treated) was maintained up to 4 h after inoculation without additional nutrient supply. However, a significant decline in the microbial load was observed after 24 h of incubation both in STW 5- and vehicle-treated samples. This drastic drop in the microbial load may be attributed to the depletion of nutrient levels in the static batch culture, or other unknown factors (32, 33). Therefore, incubation for up to 24 h may not be meaningful, and in future experiments, intermediate samples should be taken for better clarity. Due to the significant drop in absolute microbial load at tp_24_, this time point was not considered in further analyses.

The use of a substrate-limited batch culture can be regarded as a limitation of this study, since the colon can be essentially regarded as a continuous culture.

A second limitation is that this study was performed as a pilot investigation with a relatively small number of donor samples. Due to the strictness of the exclusion criteria, it was only possible to recruit six IBS patients during the study period, while for the non-IBS group, 10 donors were recruited.

Moreover, the experimental setup of the simulated upper intestinal tract digestion as well as of the gut microbial fermentation did not allow to consider absorption processes such as the uptake by intestinal transporters which may be relevant to certain STW 5 constituents under *in vivo* conditions (34, 35). Also, potential interactions of STW 5 with microbiota occurring in the small intestine, which may on the one hand be a relevant factor in the pathogenesis of IBS and may on the other hand play a role for the biotransformation of STW 5 constituents (36) remained unconsidered in the applied experimental setup. *In vivo* studies are necessary to consider these aspects in the future.

While some research reports indicate that the gut microbiome may play a role in irritable bowel syndrome, evidence remains inconclusive. Several studies have shown variations in microbial abundance and composition based on health status, with fecal samples from IBS patients generally displaying lower alpha diversity and specific microbial markers compared to those of healthy individuals (11–13, 43). Putative microbial changes appear to be influenced by the subtype and severity of IBS (11, 12). In contrast to those studies, other research observed a similar or only modestly altered microbial community of healthy and IBS subjects and concluded that poor self-rated health or reduction of the data complexity by bioinformatic methods or by selecting only the most important parameters had led to observation of differences in microbial richness and taxa between the groups (38, 43). Consistent with the latter findings, our study was unable to distinguish the HFS of IBS and non-IBS subjects based on microbiome composition. Just a trend toward lower alpha diversity in IBS stool samples was visible, which was not statistically significant. Since this study did not find any significant differences in the gut microbiome between healthy individuals and those with IBS, it suggests that any potential treatment could be equally effective for both groups. Furthermore, this enabled us to combine the data sets of both groups for further analyses.

Major compound classes present in STW 5 include phenolic compounds such as flavonoid glycosides, dihydroxycinnamic acid derivatives, and triterpene glycosides (30). These compound classes are known to have a modulatory effect on the composition and function of the human gut microbiome: the chemical structures of polyphenols suggest antimicrobial effects which may shape gut microbial community composition, but more importantly, several polyphenol classes have been shown to possess prebiotic-like effects *in vitro*, in preclinical and in clinical studies. These effects may be beneficial in diseases associated with gut microbiome dysbiosis (39). Recent findings suggest that also triterpene glycosides possess prebiotic-like effects (40). Moreover, certain microbial taxa are able to use the sugar moiety of various plant glycosides as energy source, leading on the one hand to the cleavage of these glycosides, and on the other hand to a preferential growth of these taxa (41, 42). These data suggest a potential impact of the studied preparation on the gut microbial composition.

However, in contrast to two animal studies suggesting that STW 5 affects gut microbiome composition (27, 44), only minor differences in microbial signatures were observed between STW 5- and vehicle-treated HFS in this study. At the phylum and genus levels, a small number of microbial signatures were identified as potential biomarkers for one of the additives. Interestingly, more taxa were associated with the VEH additive (10 genera) compared to STW 5 (one genus) and also the changes over time were similar between STW 5- and VEH-treated samples. This suggests that there may be an inhibitory effect of STW 5 on the decreased taxa, possibly due to antimicrobial effects of the contained polyphenols, but a comparable effect on the microbiome of both health groups.

Compared to that, STW 5-II, a formulation containing six of the nine herbs making up STW 5, has shown more pronounced effects on microbiome composition of healthy donors in recently performed *in vitro* study in a short-term colon model. This may be due to differences in experimental setup as well as in the applied formulation (31).

Consistently with their similar microbial profiles, IBS and non-IBS samples were also functionally similar in terms of biotransformation of STW 5 constituents, albeit with slightly but in most cases not significantly different biotransformation kinetics. In both groups, levels of the genuine constituents of pre-digested STW 5 were significantly decreased over incubation time in all donor samples, and this decrease paralleled an increase in the levels of their respective metabolites. Similar metabolic reactions have also been observed when STW 5-II, containing six of the nine herbs contained in STW 5, was incubated with microbiota from healthy human subjects in a short-term colonic *in vitro* model (31).

When STW 5 was incubated with the incubation medium in the absence of fecal samples, its constituents remained stable. This suggests that the fecal microbiota were responsible for the observed biotransformations.

Many classes of secondary plant metabolites have been shown previously to be biotransformed by the human gut microbiota, among them flavonoids (37, 45), hydroxycinnamic acid derivatives (46, 47), and triterpene glycosides (40, 48), which also constitute major compound classes present in STW 5.

It has been demonstrated that certain taxa present in the human gut microbiome are capable of performing the metabolic turnover reactions observed herein. The O-deglycosylation of glycyrrhizic acid into 18β-glycyrrhetinic acid (Fig. 5A1) is accomplished by taxa possessing β-glucuronidase activity, including *Eubacterium* sp., *Ruminococcus* sp., *Streptococcus* sp., *Staphylococcus* sp., or *Clostridium* sp. (49–53).

The O-deglycosylation of flavonoids, such as the conversion of liquiritin pentosides to liquiritin observed herein (Fig. 5A2), is generally accomplished by a wide range of microbial taxa, such as *Bacteroides* sp., *Parabacteroides* sp., *Lactobacillus* sp., *Bifidobacterium* sp., *Enterococcus* sp., and *Eubacterium* sp. (37). The further biotransformation of liquiritin to the dihydrochalcone davidigenin constitutes a reductive cleavage of the ether bond in ring C. This conversion has been described to be accomplished by an oxygen-sensitive NADH-dependent flavanone- and flavanonol-cleaving reductase from the human intestinal anaerobe *Eubacterium ramulus* (54). Metabolism of the 2-glucosyloxy-4-methoxycinnamic acid isomer starts with deglycosylation, followed on one hand by formation of a δ-lactone ring, leading to intermittently increased herniarin, and on the other hand by reduction of the exocyclic double bond, leading to 2-(2-hydroxy-4-methoxyphenyl) propionic acid that increased over time and may additionally result from further conversion of herniarin (Fig. 5A3). While the lactonization of (Z)−2-glucosyloxy-4-methoxycinnamic acid to herniarin has been described in plants (55), it is hitherto unknown to be accomplished by gut microorganisms. 2-(2-Hydroxy-4-methoxyphenyl) propionic has been observed as gut microbial metabolite of herniarin in rats (56). However, the microorganisms and enzymes involved in its formation which includes hydrolysis of the lactone ring and hydrogenation of the exocyclic double bond have not been discovered to date.

The only detectable metabolite potentially derived from the microbial biotransformation of rosmarinic acid was 3-(3-hydroxyphenyl)propanoic acid (Fig. 5A4). Esterases capable to hydrolyze rosmarinic acid (57) and other hydroxycinnamic acid esters (58, 59) are known from various intestinal microorganisms, including *Lactobacillus* sp., *Bifidobacterium* sp., and *Escherichia* sp. Subsequent hydrogenation of the exocyclic double bond and dehydroxylation in the aromatic ring lead to the formation of the observed 3-(3-hydroxyphenyl)propanoic acid (47). In an early study, *Peptostreptococcus* spp. and *Clostridium perfringens* were found capable to hydrogenate caffeic acid, and *E. coli* and *Steptococcus faecalis* var. *liquefaciens* were able to dehydroxylate dihydrocaffeic acid (60). Studies on involved enzymes are not available to date.

In the present study, we did not observe any significant changes in the relative abundance of any of these taxa or identify any of them as characteristics microbial signatures for STW 5-treated HFS. Moreover, contrary to our presumptions, the taxa characteristic for pre-digested VEH via MaAsLin analysis were not negatively correlated with the STW 5 constituents or their metabolites. Hence, even though we could detect the turnover of the STW 5 constituents into their major microbiome-induced metabolites, we were not able to identify the responsible taxa. This may have several reasons: first, in the substrate-limited batch culture where no nutrients had been added, with just the remaining nutrients from the fecal suspension available, STW 5 constituents might not have been sufficient to allow survival and/or replication of the present microbes, thus leading to only minor and insignificant microbial shifts in the incubates; second, possibly the taxa responsible for the observed biotransformation reactions did not increase in their relative abundance, e.g., because the biotransformation was accomplished by microbial exoenzymes; and third, the biotransformations possibly have been accomplished by a panel of several microbial taxa with the same functional capabilities, which are therefore difficult to trace back by means of correlation analysis. Future studies should therefore include functional analyses, preferably on RNA level, to delineate the responsible microbial enzymes and taxa, upregulation of which would be expected.

Overall, our results indicate that despite STW 5 addition did not significantly affect the fecal microbiome composition, major constituents of STW 5 that are possibly able to reach the colon were extensively biotransformed by fecal bacteria, regardless of the donor’s health status, to form breakdown products. The metabolites originating from the constituents of STW 5 may subsequently have the potential to elicit pharmacological effects. For example, davidigenin, the metabolite resulting from biotransformation of liquiritin pentosides, has been shown to possess anti-inflammatory and spasmolytic activity *in vitro* and *ex vivo* (61, 62). Also, for 3-(3-hydroxyphenyl)propanoic acid and 18β-glycyrrhetinic acid, the metabolites resulting from glycyrrhizic acid and rosmarinic acid biotransformation, anti-inflammatory and cytoprotective effects have been described (63–65). These bioactive metabolites may contribute to the beneficial effects observed for STW 5 in IBS.

In this study, we were able to show that microbial biotransformation of STW 5 constituents into potentially bioactive metabolites occurs fast, but independent from large microbial community shifts and independent from the health status of the fecal sample donors. The fact that also gut microbiota from IBS donors are able to produce these metabolites indicates that IBS patients may profit from their potential health-beneficial effects.

## MATERIALS AND METHODS

### Reagents

Disodium hydrogen phosphate (9J011450) was purchased from PanReac AppliChem ITW Reagents. Sodium chloride (27810.262), potassium chloride (26764.260), and methanol (UHPLC use, 20864.320) were purchased from VWR. Potassium dihydrogen phosphate (1.04873.1000) and sodium hydroxide pellets (1.06498.0500) were obtained from Merck KGaA. Alfa Aesar supplied L-cysteine HCl (anhydrous 98%; L06327). PMA (20 mM) was acquired from Biotium.

### Herbal medicinal product information

Lyophilized STW 5 (dry residue 5.71%, batch number 631714) was kindly provided by Steigerwald Arzneimittel GmbH (Bayer Consumer Health). The quality of this batch complies with the quality prerequisites for STW 5 as described by reference (66). The preparation was pre-digested in a static three-phase *in vitro* digestion model called InfoGest (30). After the intestinal phase of pre-digestion, the final pre-digested STW 5, as well as the pre-digested vehicle, was composed of a mixture of STW 5 constituents (vehicle = 0.625% ethanol for pre-digested vehicle), fluids of all three phases (simulated salivary, stomach, and small intestine fluid), the enzymes pepsin and pancreatin, and bile acids (30).

STW 5 contains a unique combination of herbal extracts: fresh plant extract of bitter candytuft (*Iberis amara* L.) and extracts from eight dried herbs, including angelica roots (*Angelica archangelica* L.), chamomile flowers (*Matricaria chamomilla* L.), caraway fruit (*Carum carvi* L), milk thistle fruit (*Silybum marianum* L.), lemon balm leaves (*Melissa officinalis* L.), peppermint leaves (*Mentha x piperita* L.), greater celandine herb (*Chelidonium majus* L.), and licorice root (*Glycyrrhiza glabra* L.).

### Preparation of pre-digested study samples

*In vitro* digestion of lyophilized STW 5 was performed as previously described (30), based on the slightly modified Infogest protocol (67), a standardized static *in vitro* model to simulate upper intestinal tract digestion. Briefly, a solution of lyophilized STW 5 in 5% (vol/vol) ethanol (6.96 mg/mL) was subsequently subjected to oral phase digestion with simulated salivary fluid containing no α-amylase, to gastric phase digestion, involving incubation with simulated gastric fluid containing pepsin, and to intestinal phase digestion, i.e., incubation with simulated intestinal fluid containing pancreatin and ox bile. In every digestive phase, the sample was diluted 1:1, resulting in a final STW 5 concentration of 0.87 mg/mL. In parallel, a control sample (VEH) was prepared by subjecting 5% (vol/vol) aqueous ethanol to the same incubation procedure. The incubation experiment was performed in 10 replicates for STW 5 and for VEH, respectively. The intestinal phase incubates of STW 5 and the control sample (VEH) (ca. 80 mL per replicate) were pooled, divided into 40 mL of aliquots, and kept frozen at −80°C prior to being subjected to incubation with fecal suspensions.

### Ethics vote and recruitment of donors

All experiments were performed in accordance with the relevant guidelines and regulations. The protocol for the use of human stool samples was approved by the Ethics Commission at Medical University of Graz (reference number: 30-451 ex 17/18) and was in accordance with the Helsinki Declaration of 1975. Written informed consent was obtained from all patients.

For this study, we recruited 6 patients suffering from IBS (ROME IV criteria; diarrhea-predominant type) and 10 non-IBS patients. The exclusion criteria for donor recruitment were BMI > 30, smoker, younger than 18 or older than 60 years of age, colonoscopy in the last 3 months, intestinal infection in the last 3 months, gastro-intestinal resection (except appendectomy), antibiotics in the last 3 months, probiotics in the last month, permanent medication intake (except thyroid preparations), proton-pump inhibitor intake, STW 5 intake in the last month, travels beyond Europe in the last month, and vegetarian or vegan diet.

IBS was diagnosed by the clinicians C.H. and P.K. according to the ROME IV criteria (68). The study participants answered a questionnaire written in German in order to verify whether the criteria for the subject’s recruitment concur with the exclusion criteria. In addition, a drinking and diet plan was recorded for 3 days before defecation. The IBS symptom severity was determined by the IBS severity scoring system (IBS-SSS) (69). An IBS-SSS of 197 was calculated. Thus, the IBS group had an average severity of moderate (five with moderate severity and one donor with a mild form of IBS). The IBS group and non-IBS group were comparable with respect to age, BMI, gender distribution, and defecation frequency (unpaired, two-sided Student’s *t* test; *P* < 0.05; Table S3). IBS patients more often suffered from gastro-intestinal complaints and intolerances than the non-IBS group (Table S3). The data from the recorded dietary questionnaires indicated that patients suffering from IBS avoided fat- and sugar-rich foods, as well as soft drinks and coffee (Table S4). In retrospect, three donors of the IBS group exhibited divergent criteria; BMI > 30 kg/m^2^ (=BMI of 30.5), age <18 years (=17 years), and one donor underwent non-documented trazodone therapy.

### Incubation experiment

1× PBS buffer, adjusted to pH 7.4, was de-oxygenated by bubbling with N_2_ for 30 min, reduced with 0.4 g L-cysteine HCl, and subsequently autoclaved. In order to prepare 11.11% (wt/vol) HFS, the freshly passed feces was transferred to an anaerobic chamber (Don Whitley A85; 37°C; gas phase 80% N_2_, 10% H_2_, and 10% CO_2_) and mixed with the sterile, anoxic PBS buffer as described previously (70). Homogeneity was obtained by manually agitating and mixing with a magnetic stirrer in a water bath at 37°C.

For the incubation experiment, 27 mL of 11.11% HFS was mixed with 3 mL pre-digested STW 5 or pre-digested vehicle in five replicates under anaerobic conditions at 37°C to achieve a final concentration of 10% HFS. To rule out degradation by the incubation conditions, the stability of pre-digested STW 5 during incubation in anoxic PBS buffer containing no fecal suspension was assessed by incubating 27 mL anoxic PBS buffer with 3 mL pre-digested STW 5 or pre-digested vehicle in five replicates (70). The final concentration 0.087 mg of STW 5 lyophilizate per mL of a 10% HFS was based on the daily dosage of STW 5 (corresponds to 171.3 mg lyophilizate) and an estimated small intestine fluid volume of approximately 200 mL (30). Samples were collected after 30 min (tp_0.5_), 4 h (tp_4_), and 24 h (tp_24_).

### Microbiome analysis—DNA extraction, microbial load and composition

For microbiome analysis, 250 µL of the drawn samples was immediately treated with 1.25 µL PMA to evaluate the abundance of viable bacterial cells present at this time point. After incubation without light exposure for 5 min (IKA Rocker 2D digital) and subsequent light treatment (PMA-Lite LED Photolysis Device; Biotium) for 15 min, samples were frozen at −80°C and stored until analysis. HFS microbiome control samples (c_0Mic_) without any additive (vehicle or STW 5) were prepared in the same way. DNA was extracted from all samples containing HFS (in total 560 samples from time points c_0Mic_, tp_0.5_, tp_4_, and tp_24_) using the E.Z.N.A. Stool DNA Kit (Omega bio-tek) according to the manufacturer’s extraction protocol.

#### Absolute abundance

For quantification, Sso Advanced Universal SYBR Green Supermix (Bio-Rad) was used together with the following primer pair, forward primer 331F (5′-TCCTACGGGAGGCAGCAGT-3′) and reverse primer 797R (5′-GGACTACCAGGGTATCTAATCCTGTT-3′) (71). PCR reactions were carried out in a CFX384 Touch Real-time PCR Detection System (Bio-Rad). Each sample was analyzed in triplicates. After an initial denaturation at 95°C for 15 min, the following cycling conditions were repeated 40 times: 94°C for 15 s (denaturation), 54°C for 30 s (annealing), 72°C for 40 s (elongation + plate read). The efficiency of all runs was >90%. The detection limit was defined by the average Cq values of our non-template controls. The quantity of each sample was given in microbial load per gram of stool.

#### Relative abundance

16S rRNA-targeted Illumina Next-Generation Sequencing was performed for all collected samples. To amplify the 16S rRNA gene V4 hypervariable region, the following primers: F515 (5′-TCGTCGGCAGCGTCAGATGTGTATAAGAGACAGGTGCCAGCMGCCGCGGTAA-3′) and R806 (5′-GTCTCGTGGGCTCGGAGATGTGTATAAGAGACAGGGACTACHVGGGTWTCTAAT-3′) (72). The reaction conditions were: 94°C for 3 min (initial denaturation), followed by 32 cycles of 94°C for 45 s (denaturation), 50°C for 60 s (annealing), and 72°C for 90 s (elongation), and 72°C for 10 min (final elongation). Extraction blanks and PCR negative controls were processed in parallel. The amplicons were sequenced by the Illumina MiSeq technique, which was performed in cooperation with the Core Facility Molecular Biology at the Center for Medical Research at the Medical University of Graz, Austria (73).

In short, the resulting fastq files were analyzed using Quantitative Insights Into Microbial Ecology (QIIME2) (74), which includes the Divisive Amplicon Denoising Algorithm (DADA2) (75). Paired end reads were joined and the quality of the produced sequences was checked. Representative sequences were taxonomically classified using SILVA v132 as the reference database (76). The R package decontam (77) was used to deal with potential contaminations (threshold 0.5 and prevalence method). Subsequently, features belonging to mitochondria and chloroplasts were removed together with features with 0 reads. SRS (scaling with ranked subsampling; https://vitorheidrich.shinyapps.io/srsshinyapp/) normalization was used to set a minimum sampling depth of 10,000 reads per sample.

### Statistical analysis and data visualization of the microbial information

The final RSV table was used to perform different microbiome analyses using the web-based tool NAMCO (78) (including alpha and beta diversity) and R studio. The packages used for the different analyses and visualizations are as follows: “Maaslin2” (differential abundance analysis; default settings); “ggpubr” and “tidyverse” (Spearman’s rho correlation and heatmaps); “ggpubr,” “tidyverse,” “dplyr,” and “scales” (most abundant taxa). Furthermore, an overview of the most abundant taxa was also generated using the web-based tool RAWgraphs (79).

Statistical analyses were performed with IBM SPSS Amos version 27 and the effect size of the PCoA plots was calculated using the R packages “phyloseq,” phytools,” and “vegan.”

### Ultra high-performance liquid chromatography-mass spectrometry metabolomics analysis and data processing

For UHPLC-HRMS analysis, the HFS and PBS buffer samples were centrifuged at 13,000 rpm at 4°C for 10 min, filtrated (0.45 µm), aliquoted, and stored at −20°C until utilization. In addition, metabolomic control samples (c_0Met_) were prepared by mixing centrifuged and filtrated HFS with pre-digested STW 5 or vehicle at a 10:1 ratio.

Before UHPLC-HRMS, the samples were thawed, vortexed three times for 10 s, and centrifuged at 13,000 rpm for 10 min at room temperature. In addition, we measured a blank with pure methanol. For UHPLC-HRMS analysis, we used the same method described in (30).

The LCMS data for each donor were processed using Compound Discoverer 2.1, separately in negative and positive mode as described previously (30), with the following modifications: Total intensity threshold was 100,000 for spectrum selection and detection of unknown compounds. RT tolerance for grouping was 0.3 min. The resulting feature table was exported to Microsoft Excel, where mean peak areas of the five technical replicates were calculated and areas were compared between STW 5- and vehicle-treated samples. Features with ratios >150% of the HFSs from at least one of the donors or time points, as well as features with ratios >150% in the PBS buffer samples without HFS were considered to be related to STW 5 or its metabolites and kept for further data evaluation. In order to calculate the metabolization ratios of the genuine STW 5 constituents and the newly formed metabolites at each time point (tp_0.5_ and tp_4_), ratios were set in relation to the c_0Met_ samples (i.e., microbiota-free control samples in which STW 5 was added after centrifugation and filtration of the fecal suspensions). Significantly different features (*P* < 0.05; peak areas and metabolization ratios: tp_0.5_/c_0Met_ or tp_4_/c_0Met_) were determined using ALDEx2 (80). PBS-buffer control incubations for pre-digested STW 5 and its vehicle were also prepared for the same time points (tp_0.5_ or tp_4_). The respective peak areas of the detected STW 5 constituents were compared to the peak areas in the microbiome-free contol samples to differentiate metabolization processes induced by gut microbiota from those induced by other factors, such as incubation temperature and time (70).

Due to the low concentration of STW 5 used in the incubation experiments (0.087 mg/mL) in order to stay in the range of the recommended daily dose of the preparation, and due to the interference of highly abundant matrix constituents derived from *ex vivo* pre-digestion and from the fecal samples, only the major STW 5 constituents were detectable in c_0Met_ samples and therefore annotated. Annotation of STW 5 constituents and metabolites was either performed by comparison with authentic references or based on reference data in the literature.

## Data Availability

The authors confirm that the data supporting the findings of this study are available within the article and its supplementary materials. The 16S rRNA sequencing data in this study have been deposited at EMBL-ENA (PRJEB42636). The LCMS raw data have been deposited at MetaboLights under accession number MTBLS2398.

## SUPPLEMENTAL MATERIAL

Supplemental material spectrum.04031-23-s0001.pdf Fig. S1 to S3; Tables S2 to S4.

Supplemental table spectrum.04031-23-s0002.xlsx Table S1.

ASM does not own the copyrights to Supplemental Material that may be linked to, or accessed through, an article. The authors have granted ASM a non-exclusive, world-wide license to publish the Supplemental Material files. Please contact the corresponding author directly for reuse.
