# Supplementary material for: Rapid biotransformation of STW 5 constituents by human gut microbiome from IBS- and non-IBS donors
Source: Microbiol Spectr. 2024 May 13;12(6):e04031-23. doi: 10.1128/spectrum.04031-23 (PMC11237759; doi:10.1128/spectrum.04031-23)
Supplement: Supplemental material — Fig. S1 to S3; Tables S2 to S4. [file spectrum.04031-23-s0001.pdf]

Rapid biotransformation of STW 5 constituents by human gut microbiome from IBS and non-IBS donors

Timo A. Thumann[a,b], Eva-Maria Pferschy-Wenzig[a,b], Christina Kumpitsch[c], Stefanie Duller[c], Christoph Högenauer[d], Patrizia Kump[d], Heba Aziz-Kalbhenn[e], Ramy M. Ammar[e,f], Sabine Rabini[e], Christine Moissl-Eichinger[b,c], Rudolf Bauer[a,b,#]

[a]Institute of Pharmaceutical Sciences, Department of Pharmacognosy, University of Graz, Beethovenstraße 8, 8010 Graz, Austria
[b]BioTechMed, Mozartgasse 12, 8010 Graz, Austria
[c]Diagnostic and Research Institute of Hygiene, Microbiology and Environmental Medicine, Medical University of Graz, Neue Stiftingtalstraße 6, 8010 Graz, Austria
[d]Department of Internal Medicine, Medical University of Graz, Auenbruggerplatz 15, 8036, Graz, Austria
[e]Steigerwald Arzneimittelwerk GmbH, Bayer Consumer Health, Havelstraße 5, 64295 Darmstadt, Germany
[f]Department of Pharmacology, Faculty of Pharmacy, Kafrelsheikh University, 33511 Kafrelsheikh; Egypt

Running title: STW 5 constituents and human gut microbiome

[#]Address correspondence to Rudolf Bauer, rudolf.bauer@uni-graz.at

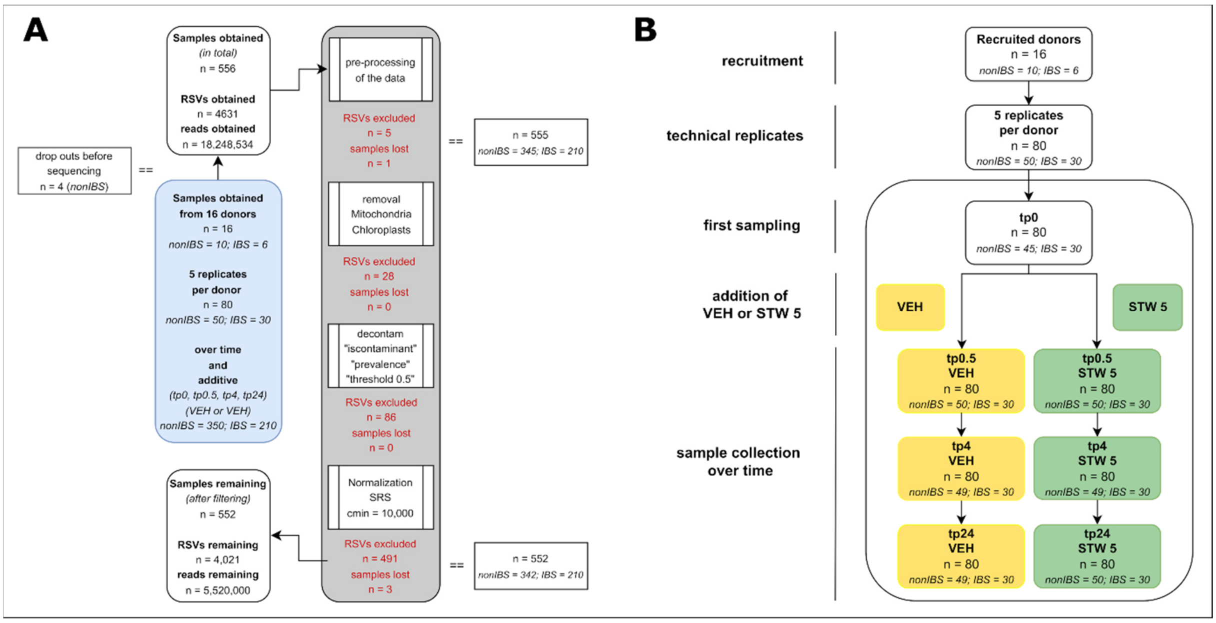

Figure S1. Analytical sample size flowchart of the study. **A.** Information on the overall sample size, filter settings and dropouts of the study. **B.** Actual number of samples per group (time point, additive) analyzed in this study.

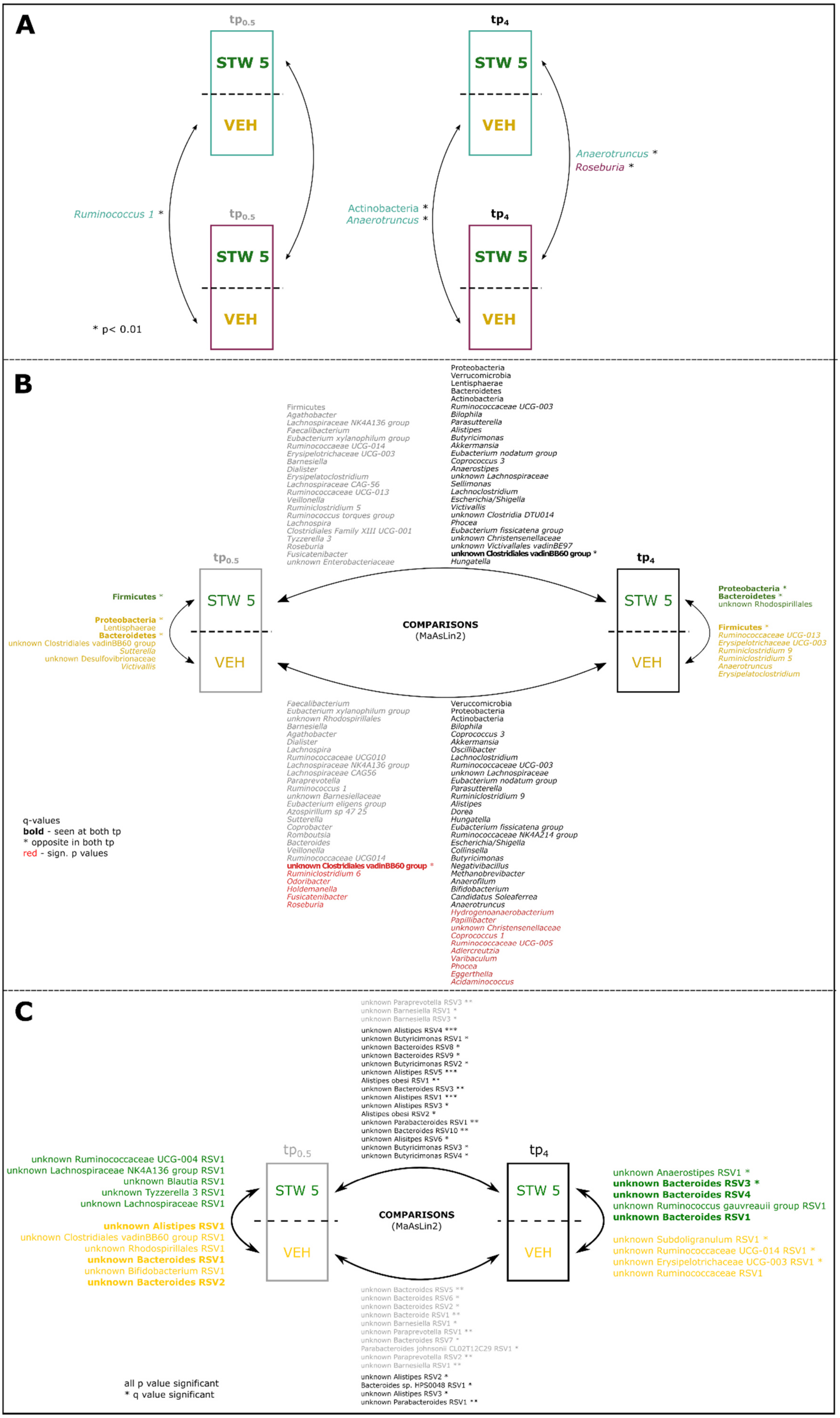

A
tp0.5
STW 5
VEH
tp4
STW 5
VEH
Anaerotruncus *
Roseburia *
Ruminococcus 1 *
Actinobacteria *
Anaerotruncus *
tp0.5
STW 5
VEH
tp4
STW 5
VEH
* p< 0.01
B
Firmicutes
Agathobacter
Lachnospiraceae NK4A136 group
Faecalibacterium
Eubacterium xylanophilum group
Ruminococcaeae UCG-014
Erysipelotrichaceae UCG-003
Barnesiella
Dialister
Erysipelatoclostridium
Lachnospiraceae CAG-56
Ruminococcaceae UCG-013
Veillonella
Ruminiclostridium 5
Ruminococcus torques group
Lachnospira
Clostridiales Family XIII UCG-001
Tyzzerella 3
Roseburia
Fusicatenibacter
unknown Enterobacteriaceae
Proteobacteria
Verrucomicrobia
Lentisphaerae
Bacteroidetes
Actinobacteria
Ruminococcaceae UCG-003
Bilophila
Parasutterella
Alistipes
Butyricimonas
Akkermansia
Eubacterium nodatum group
Coprococcus 3
Anaerostipes
unknown Lachnospiraceae
Sellimonas
Lachnoclostridium
Escherichia/Shigella
Victivallis
unknown Clostridia DTU014
Phocea
Eubacterium fissicatena group
unknown Christensenellaceae
unknown Victivallales vadinBE97
unknown Clostridiales vadinBB60 group *
Hungatella
tp0.5
STW 5
VEH
tp4
STW 5
VEH
Firmicutes *
Proteobacteria *
Lentisphaerae
Bacteroidetes *
unknown Clostridiales vadinBB60 group
Sutterella
unknown Desulfovibrionaceae
Victivallis
COMPARISONS
(MaAsLin2)
Proteobacteria *
Bacteroidetes *
unknown Rhodospirillales
Firmicutes *
Ruminococcaceae UCG-013
Erysipelotrichaceae UCG-003
Ruminiclostridium 9
Ruminiclostridium 5
Anaerotruncus
Erysipelatoclostridium
Faecalibacterium
Eubacterium xylanophilum group
unknown Rhodospirillales
Barnesiella
Agathobacter
Dialister
Lachnospira
Ruminococcaceae UCG010
Lachnospiraceae NK4A136 group
Lachnospiraceae CAG56
Paraprevotella
Ruminococcus 1
unknown Barnesiellaceae
Eubacterium eligens group
Azospirillum sp 47 25
Sutterella
Coprobacter
Romboutsia
Bacteroides
Veillonella
Ruminococcaceae UCG014
unknown Clostridiales vadinBB60 group *
Ruminiclostridium 6
Odoribacter
Holdemanella
Fusicatenibacter
Roseburia
Veruccomicrobia
Proteobacteria
Actinobacteria
Bilophila
Coprococcus 3
Akkermansia
Oscillibacter
Lachnoclostridium
Ruminococcaceae UCG-003
unknown Lachnospiraceae
Eubacterium nodatum group
Parasutterella
Ruminiclostridium 9
Alistipes
Dorea
Hungatella
Eubacterium fissicatena group
Ruminococcaceae NK4A214 group
Escherichia/Shigella
Collinsella
Butyricimonas
Negativibacillus
Methanobrevibacter
Anaerofilum
Bifidobacterium
Candidatus Soleaferrea
Anaerotruncus
Hydrogenoanaerobacterium
Papillibacter
unknown Christensenellaceae
Coprococcus 1
Ruminococcaceae UCG-005
Adlercreutzia
Varibaculum
Phocea
Eggerthella
Acidaminococcus
q-values
bold - seen at both tp
* opposite in both tp
red - sign. p values
C
unknown Paraprevotella RSV3 **
unknown Barnesiella RSV1 *
unknown Barnesiella RSV3 *
unknown Alistipes RSV4 ***
unknown Butyricimonas RSV1 *
unknown Bacteroides RSV8 *
unknown Bacteroides RSV9 *
unknown Butyricimonas RSV2 *
unknown Alistipes RSV5 ***
Alistipes obesi RSV1 **
unknown Bacteroides RSV3 **
unknown Alistipes RSV1 ***
unknown Alistipes RSV3 *
Alistipes obesi RSV2 *
unknown Parabacteroides RSV1 **
unknown Bacteroides RSV10 **
unknown Alisitpes RSV6 *
unknown Butyricimonas RSV3 *
unknown Butyricimonas RSV4 *
unknown Ruminococcaceae UCG-004 RSV1
unknown Lachnospiraceae NK4A136 group RSV1
unknown Blautia RSV1
unknown Tyzzerella 3 RSV1
unknown Lachnospiraceae RSV1
unknown Alistipes RSV1
unknown Clostridiales vadinBB60 group RSV1
unknown Rhodospirillales RSV1
unknown Bacteroides RSV1
unknown Bifidobacterium RSV1
unknown Bacteroides RSV2
tp0.5
STW 5
VEH
COMPARISONS
(MaAsLin2)
tp4
STW 5
VEH
unknown Anaerostipes RSV1 *
unknown Bacteroides RSV3 *
unknown Bacteroides RSV4
unknown Ruminococcus gauvreauii group RSV1
unknown Bacteroides RSV1
unknown Subdoligranulum RSV1 *
unknown Ruminococcaceae UCG-014 RSV1 *
unknown Erysipelotrichaceae UCG-003 RSV1 *
unknown Ruminococcaceae RSV1
unknown Bacteroides RSV5 **
unknown Bacteroides RSV6 *
unknown Bacteroides RSV2 *
unknown Bacteroide RSV1 **
unknown Barnesiella RSV1 *
unknown Paraprevotella RSV1 **
unknown Bacteroides RSV7 *
Parabacteroides johnsonii CL02T12C29 RSV1 *
unknown Paraprevotella RSV2 **
unknown Barnesiella RSV1 **
unknown Alistipes RSV2 *
Bacteroides sp. HPS0048 RSV1 *
unknown Alistipes RSV3 *
unknown Parabacteroides RSV1 **
all p value significant
* q value significant

Figure S2. ***Characteristic microbial signatures (q < 0.05) detected for the different additive groups over time.*** Yellow color indicates VEH, green color indicates STW 5. Comparisons based on **A.** health status and additive group at phylum and genus level. Turquoise color indicates healthy group, purple color indicates IBS group. **B.** additive group at phylum and genus level using the combined data set. **C.** based on additive groups at RSV level using the combined data set. All analyses were performed using the SRS normalized data set. (for p-values see **Table S1_D**)

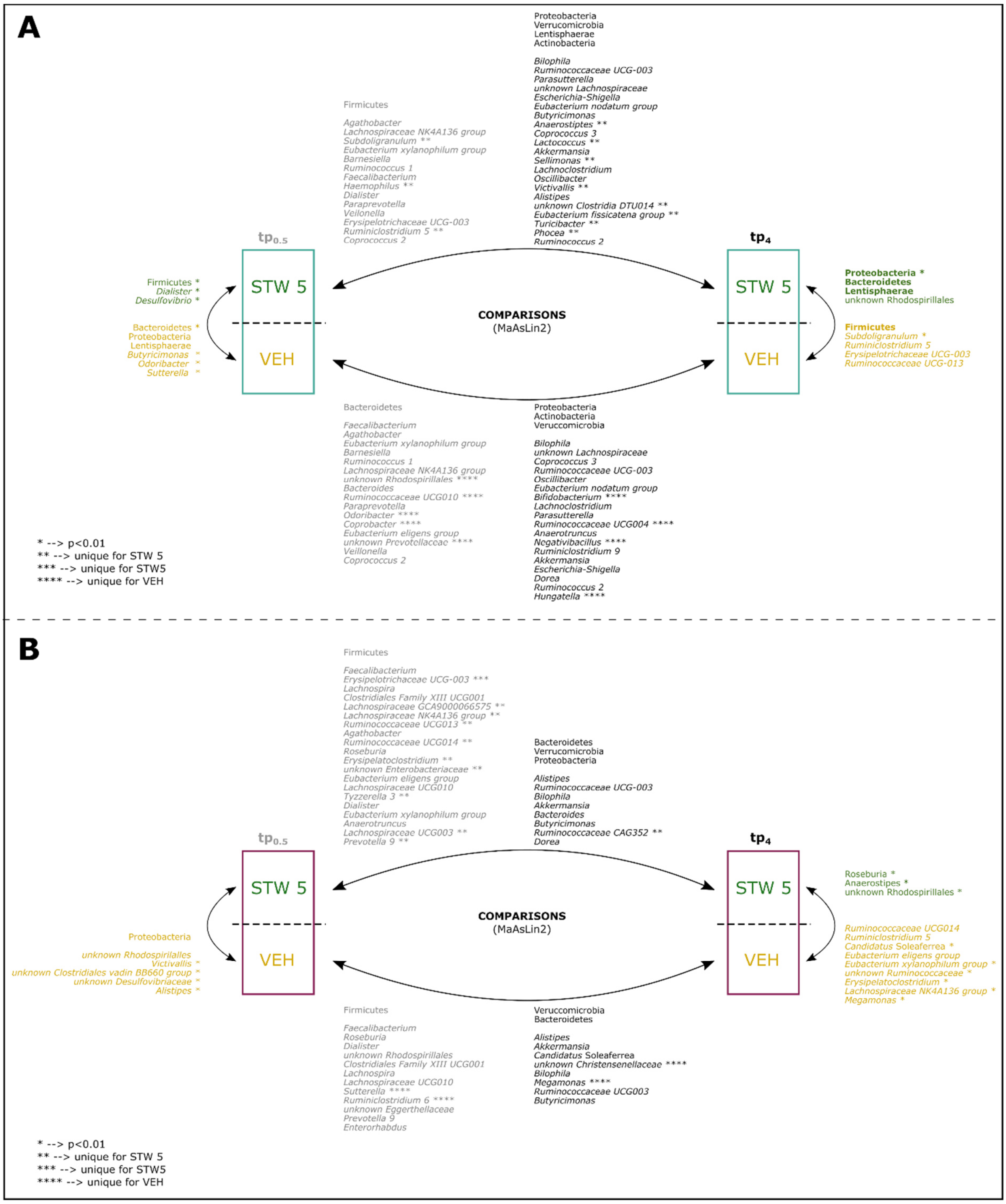

Figure S3. **Characteristic microbial signatures for STW5 and VEH within the health conditions over time.** (phylum and genus level). Yellow color indicates VEH, green color indicates STW 5. Turquoise color indicates healthy group, purple color indicates IBS group. **A:** Healthy group. **B:** IBS group. *(*for p-values see **Table S1_D***)*

Table S2. *Annotation of STW 5 constituents and metabolites.*

Compound	mode	RT (min)	Neutral mono-isotopic mass	*m/z*	dd MS^2 fragments	Neutral formula	Δ (ppm)	ID source	ID level
**Glycyrrhizic acid**	-	Identified as glycyrrhizic acid (#51) by retention time plus fragmentation pattern according to Thumann et al. (2020) and reference substance							b
**Rosmarinic acid**	-	Identified as rosmarinic acid (#24) by retention time plus fragmentation pattern according to Thumann et al. (2020) and reference substance							b
**2-Glucosyloxy-4-methoxycinnamic acid isomer**	-	Identified as 2-glucosyloxy-4-methoxycinnamic acid isomer (#4) by retention time plus fragmentation pattern according to Thumann et al. (2020)							a
**Liquiritin pentoside isomer**	-	Identified as liquiritin pentoside isomer (#12) by retention time plus fragmentation pattern according to Thumann et al. (2020)							a
**Liquiritigenin**	-	Identified as liquiritigenin (#29) by retention time plus fragmentation pattern according to Thumann et al. (2020) and reference substance							b
**Herniarin**	+	14.67	176.0473	177.0547	177.0543(100);133.0645(5);121.0646	$C_{10}H_8O_3$	0.45	reference substance, databases (MzCloud, The Human Metabolome Database and METLIN) and Pastírová et al. (2005)	b
**18β-Glycyrrhetinic acid**	+	45.63	470.3396	471.3466	471.3458(100);425.3421(5)	$C_{30}H_{46}O_{10}$	-0.69	reference substance, databases (MzCloud, The Human Metabolome Database and METLIN) and Farag et al. (2012)	b
**3-(3-Hydroxyphenyl) propionic acid**	-	6.38	166.0630	165.0550	165.0549(40);121.0646(100);119.0490(60);	$C_9H_{10}O_3$	2.36	reference substance and database (The Human Metabolome Database)	b
**3-(2-Hydroxy-4-methoxyphenyl) propanoic acid**	-	9.88	196.0736	195.0658	195.0658(60);177.0550(20);151.0754(100);136.0518(20)	$C_{10}H_{12}O_4$	3.15	databases (The Human Metabolome Database)	a

**Davidigenin**	-	25.10	258.0892	257.0822	257.0821(10);151.0390(100)	$C_{15}H_{14}O_4$	5.35	reference substance and Xu et al. (2013)	b

a= tentative identification based on literature and/or database; b= identification by retention time plus fragmentation pattern of an authentic reference compound; five substances have already been described as STW 5 constituents by Thumann et al. (2020); MzCloud https://www.mzcloud.org (accessed 15.11.2020); The Human Metabolome Database http://www.hmdb.ca (accessed 15.11.2020), METLIN https://metlin.scripps.edu (accessed 15.11.2020)

Table S3. *Comparison of patient characteristics in the IBS and non-IBS groups. Significance was tested by an unpaired, two-sided Student´s t-test.*

	**Non-IBS donors (n=10)**		**IBS donors (n=6)**		
	**Mean**	**SD**	**Mean**	**SD**	**p-value**
**Donor age, years**	36	11	38	11	0.717
**Height, cm**	175	8	173	10	0.736
**Weight, kg**	74	14	66	9	0.274
**BMI, kg/m^2**	24.1	3	22.3	4	0.334
**Sex**	f=70%<br>m=30%		f=66%<br>m=33%		
**Patients with regular gastrointestinal complaints**	10%		100%		
**Patients with intolerances (e.g., fructose, lactose, histamine)**	10%		50%		
**How many times per day do you defecate?**	1.3	0.8	2.0	1.1	0.237
**IBS-SSS**			197	36	

Table S4. *Summary of the food and drink plans of donors 3 days before sample donation*

		**Non-IBS donors (n=10)**										**IBS donors (n=6)**					
**Food**	Fat-rich		X		X	X	X		X	X	X						
	Protein-rich	X	X	X	X	X	X	X	X	X	X	X	X	X	X	X	X
	Carbohydrate-rich	X	X	X	X	X	X	X	X	X	X	X	X	X	X	X	X
	Fruit-rich	X				X							X	X			
	Vegetable-rich	X	X		X	X			X			X	X				X
	Sugar-rich	X	X		X		X	X	X	X	X			X		X	
**Drink**	Water	X	X	X	X	X	X	X	X	X	X	X	X	X	X	X	X
	Tea	X			X			X			X	X	X	X			
	Coffee	X	X		X	X		X	X	X				X			X
	Milk-based drinks			X		X										X	
	Soft drinks/juices		X	X		X	X	X	X	X			X			X	
	Beer			X			X	X									
	Wine-based drinks	X	X						X				X	X			

X indicates the donors consumed the food or drink either at more than two meal times during the 3 days, or two different food or drinks at one meal time. Fat-rich foods included butter, fried meat, butter croissant, leberkäse, fries, etc.; protein-rich foods included yoghurt, chicken, meat in general, sausages, eggs, cheese, etc.; carbohydrate-rich foods included rice, potatoes, noodles, bread, sandwiches, and gnocchi, etc.; tomato was categorized under vegetables; sugar-rich foods included cakes in all variations, cookies, ice-cream, honey, and jam.
